# Microarray data analysis to identify crucial genes regulated by *CEBPB* in human SNB19 glioma cells

**DOI:** 10.1186/s12957-016-0997-z

**Published:** 2016-10-06

**Authors:** Chenghua Du, Pan Pan, Yan Jiang, Qiuli Zhang, Jinsuo Bao, Chang Liu

**Affiliations:** 1 Department of Neurosurgery, The Affiliated Hospital of Inner Mongolia University for the Nationalities, Huolinhe Street No.1742, Tongliao, Inner Mongolia 028007 China; 2 Department of Hepatology, Tongliao City Hospital for Infectious Diseases, Tongliao, Inner Mongolia 028007 China

**Keywords:** Glioma, CCAAT/enhancer binding protein beta, Differentially expressed genes, Protein-protein interaction network, Transcriptional regulatory network

## Abstract

**Background:**

Glioma is one of the most common primary malignancies in the brain or spine. The transcription factor (TF) CCAAT/enhancer binding protein beta (*CEBPB*) is important for maintaining the tumor initiating capacity and invasion ability. To investigate the regulation mechanism of *CEBPB* in glioma, microarray data GSE47352 was analyzed.

**Methods:**

GSE47352 was downloaded from Gene Expression Omnibus, including three samples of SNB19 human glioma cells transduced with non-target control small hairpin RNA (shRNA) lentiviral vectors for 72 h (normal glioma cells) and three samples of SNB19 human glioma cells transduced with *CEBPB* shRNA lentiviral vectors for 72 h (*CEBPB*-silenced glioma cells). The differentially expressed genes (DEGs) were screened using limma package and then annotated. Afterwards, the Database for Annotation, Visualization, and Integrated Discovery (DAVID) software was applied to perform enrichment analysis for the DEGs. Furthermore, the protein-protein interaction (PPI) network and transcriptional regulatory network were constructed using Cytoscape software.

**Results:**

Total 529 DEGs were identified in the normal glioma cells compared with the *CEBPB*-silenced glioma cells, including 336 up-regulated and 193 down-regulated genes. The significantly enriched pathways included chemokine signaling pathway (which involved *CCL2*), focal adhesion (which involved *THBS1* and *THBS2*), TGF-beta signaling pathway (which involved *THBS1*, *THBS2*, *SMAD5*, and *SMAD6*) and chronic myeloid leukemia (which involved *TGFBR2* and *CCND1*). In the PPI network, CCND1 (degree = 29) and CCL2 (degree = 12) were hub nodes. Additionally, *CEBPB* and *TCF12* might function in glioma through targeting others (*CEBPB* → *TCF12*, *CEBPB* → *TGFBR2*, and *TCF12* → *TGFBR2*).

**Conclusions:**

*CEBPB* might act in glioma by regulating *CCL2*, *CCND1*, *THBS1*, *THBS2*, *SMAD5*, *SMAD6*, *TGFBR2*, and *TCF12*.

## Background

Glioma, which is known as one of the most common primary malignancies in the brain or spine, accounts for nearly 30 % of all brain and central nervous system tumors and 80 % of all malignant brain tumors [1, 2]. Previous researches have shown that the most important hallmarks of malignant glioma are its invasion and angiogenesis [3]. So far, researchers have indicated that glioma can be induced by neurofibromatoses and tuberous sclerosis complex [4], electromagnetic radiation [5], DNA repair genes (such as excision repair cross-complementing 1, *ERCC1*, and X-ray repair cross-complementing group 1, X*RCC1*) [6]. However, the exact molecular mechanisms of glioma were still unclear.

In the central nervous system, the neoplastic transformation can convert the neural cells into cells of mesenchymal phenotype which possess the ability of invasion and promoting angiogenesis [7, 8]. What is more, it has been identified that mesenchymal stem cells (MSC)-like properties may play a role in the tumorigenesis, invasion, and recurrence of primary glioblastoma tumors [8]. The transcription factor (TF) CCAAT/enhancer binding protein beta (*CEBPB*) is associated with the mesenchymal state of primary glioblastoma, and its expression in glioma is important for maintaining the tumor initiating capacity and invasion ability [9, 10]. Moreover, the transforming growth factor beta 1/SMAD family member 3 (*TGFB1*/*SMAD3*) plays a key role in the extracellular matrix (ECM) production which can lead to glioblastoma aggression [11, 12]. It has been revealed that *CEBPB* can regulate the synthesis of ECM [13]. However, the regulation mechanism of *CEBPB* on *TGFB1*/*SMAD3* in glioma was seldom studied.

In our study, in order to gain a better understanding of the regulation mechanisms of *CEBPB* and investigate whether *CEBPB* could regulate the production of ECM via the *TGFB1*/*SMAD3* signaling pathway in glioma, the microarray data deposited by Carro et al. were further analyzed with bioinformatics methods. Firstly, the differentially expressed genes (DEGs) between SNB19 human glioma cells transduced with non-target control small hairpin RNA (shRNA) lentiviral vectors for 72 h and SNB19 human glioma cells transduced with *CEBPB* shRNA lentiviral vectors for 72 h were identified and annotated. Subsequently, their potential functions were predicted by enrichment analysis. Finally, protein-protein interaction (PPI) network and transcriptional regulatory network were constructed to screen key genes.

## Methods

### Microarray dataset

The microarray dataset of GSE19114 [14] was downloaded from Gene Expression Omnibus (GEO, http://www.ncbi.nlm.nih.gov/geo/) database, which was based on the platform of GPL6947 IlluminaHumanHT-12 V3.0 expression beadchip. A total of 74 samples were included in the dataset, among which 3 samples of SNB19 human glioma cells transduced with non-target control shRNA lentiviral vectors for 72 h (normal glioma cells) and 3 samples of SNB19 human glioma cells transduced with *CEBPB* shRNA lentiviral vectors for 72 h (*CEBPB*-silenced glioma cells) were used to study the effect of *CEBPB* on glioma.

### Data preprocessing and DEGs screening

The preprocessed microarray data were obtained from GEO2R of National Center of Biotechnology Information (NCBI, http://www.ncbi.nlm.nih.gov/geo/geo2r/), including 48803 probes. The linear models for microarray data (limma) package [15] were used to identify the DEGs between the normal glioma cells and the *CEBPB*-silenced glioma cells. Benjamini-Hochberg (BH) method [16] was applied to adjust the raw *p* value into false discovery rate (FDR). The FDR <0.05 and |log_2_ fold change (FC) >1 were used as cut-off criteria.

### Functional and pathway enrichment analysis

Gene Ontology (GO, http://www.geneontology.org/) annotations are of great importance for mining biological and functional significance from large dataset [17]. The Kyoto Encyclopedia of Genes and Genomes (KEGG, http://www.genome.ad.jp/kegg) database represents higher order of functions in terms of the network of the interacting molecules [18]. The Database for Annotation, Visualization, and Integrated Discovery (DAVID) online tool [19] was employed to perform GO functional and KEGG pathway enrichment analyses for the DEGs. The *p* value <0.05 was used as the cut-off criterion.

### DEGs annotation

TSGene database (http://bioinfo.mc.vanderbilt.edu/TSGene/), which contains detailed annotations for each tumor suppressor gene (TSG), such as cancer mutations, gene expressions, methylation sites, transcriptional regulations, and PPIs, was applied to identify the TSGs from the DEGs [20]. Additionally, tumor-associated gene (TAG) database (http://www.binfo.ncku.edu.tw/TAG/), which provides information about commonly shared functional domains in well-characterized oncogenes and TSGs, was used for screening the TAGs from the DEGs [21]. Besides, as a collection of data about the transcriptional regulatory network, the Encyclopedia of DNA Elements (ENCODE) project was introduced for screening the TFs from the DEGs [22].

### PPI network construction

The PPI pairs were searched using the Search Tool for the Retrieval of Interacting Genes (STRING, http://string-db.org/) online tool [23]. The required confidence (combined score) >0.4 was used as the cut-off criterion. Then, the Cytoscape software [24] was used to visualize the PPI network. Furthermore, connectivity degree analysis was performed to search the hub nodes of PPI networks. The degree of a node was corresponded to the number of interactions involved it [25]. In addition, hub nodes were nodes with higher degrees.

### Transcriptional regulatory network construction

ENCODE project is a collection of data about the transcriptional regulatory network, which helps illuminate TF-binding sites, histone marks, chromatin accessibility, DNA methylation, RNA expression, RNA binding, and other cell-state indicators [22]. Based on the transcriptional regulation interactions derived from ENCODE project, the regulatory network containing *CEBPB* and *TGFB1*/*SMAD3* was constructed by Cytoscape software [24].

## Results

### Identification of DEGs

According to the analysis of the microarray dataset, a total of 529 DEGs (including 336 up-regulated genes and 193 down-regulated genes) were identified in the normal glioma cells compared with the *CEBPB*-silenced glioma cells. Among them, the top ten significantly up-regulated genes (such as thrombospondin 1 (*THBS1*) and chemokine (C-C motif) ligand 2 (*CCL2*)) and down-regulated genes (such as cyclin D1 (*CCND1*)) are displayed in Table 1.

**Table 1:** The top ten up- and down-regulated genes

DEGs	Gene symbol	FDR	Log_2_ FC
Up-regulated	*AXL*	9.39E−07	1.846031
	*SERPINE1*	8.58E−07	1.741651
	*ITGB1*	6.28E−08	1.739866
	*PRPF31*	6.28E−08	1.644503
	*TXNDC5*	3.26E−08	1.629988
	*WDFY1*	3.26E−08	1.622947
	*AXL*	1.57E−07	1.554728
	*SLC1A3*	5.96E−08	1.484443
	*SET*	3.90E−07	1.477058
	*ITGB1*	2.66E−07	1.466634
Down−regulated	*AKR1B10*	3.26E−08	−2.19537
	*SLC2A3*	6.28E−08	−2.01825
	*HMOX1*	6.28E−08	−1.58464
	*CCND1*	9.30E−08	−1.49158
	*HIST1H2BK*	1.16E−07	−1.38961
	*STX3*	3.36E−07	−1.2468
	*TDG*	8.98E−08	−1.23629
	*SRXN1*	8.97E−07	−1.22479
	*DICER1*	5.00E−07	−1.20817
	*STK40*	9.14E−07	−1.19625

*DEGs* differentially expressed genes, *FDR* false discovery rate, *FC* fold change

### Functional and pathway enrichment analysis

For the up-regulated genes, the enriched functions included transcription from RNA polymerase II promoter (*p* = 1.01E−03), cytoskeleton organization (*p* = 2.76E−04), and endocytosis (*p* = 2.57E−05) (Table 2A). Meanwhile, the down-regulated genes were mainly enriched in the function of enzyme-linked receptor protein signaling pathway (*p* = 2.89E−03), skin development (*p* = 4.97E−03), and response to hyperoxia (*p* = 2.97E−05) (Table 2B).

**Table 2:** The top ten functions enriched for the differentially expressed genes

GO ID	Description	Gene number	*p* value	Gene symbols
(A)				
GO:0006366	Transcription from RNA polymerase II promoter	47	1.01E−03	*SOX21*, *TCF25*, *TOP2A*, *GTF2F2*, *CIAO1*, *SERPINE1*, *DKK1*, *CYR61*, *SOX18*, *PAF1…*
GO:0007010	Cytoskeleton organization	32	2.76E−04	*PTK2*, *DPYSL2*, *CNN3*, *BICD2*, *CLIC4*, *CTGF*, *EDN1*, *NRAS*, *ITGB1*, *RHOG…*
GO:0006897	Endocytosis	23	2.57E−05	*PTK2*, *PIK3R2*, *THBS1*, *SERPINE1*, *DKK1*, *CYFIP2*, *AXL*, *RABEPK*, *LRP1B*, *ABCA1…*
GO:0071375	Cellular response to peptide hormone stimulus	15	5.75E−04	*PTK2*, *PIK3R2*, *GNG10*, *PPM1A*, *GNG5*, *PIK3R1*, *ATP6V1G1*, *NRAS*, *SOCS2*, *GNG12…*
GO:0000398	mRNA splicing, via spliceosome	10	1.02E−02	*PABPC1*, *GTF2F2*, *LSM7*, *LSM3*, *POLR2C*, *UPF3B*, *MBNL2*, *C1QBP*, *PRPF31*, *PAPOLA*
GO:0048469	Cell maturation	9	8.96E−04	*SOX18*, *AXL*, *GJA1*, *DLD*, *FOXO3*,*TYMS*,*CLN5*,*EPAS1*,*PTBP3*
GO:0043200	Response to amino acid stimulus	7	6.71E−04	*CTGF*, *EDN1*, *CEBPB*, *TYMS*, *CCL2*, *LAMTOR3*, *LAMTOR1*
GO:0006112	Energy reserve metabolic process	7	4.38E−02	*GNG10*, *GNG5*, *GFPT2*, *RAP1B*, *PPP1CC*, *GNG12*, *PYGB*
GO:0018279	Protein N-linked glycosylation via asparagine	6	1.02E−02	*UGGT1*, *MLEC*, *GFPT2*, *B4GALT5*, *PGM3*, *STT3B*
GO:0006261	DNA-dependent DNA replication	6	1.49E−02	*POLB*, *MCM3*, *RFC5*, *TOP2A*, *BAZ1A*, *RPAIN*
(B)				
GO:0007167	Enzyme-linked receptor protein signaling pathway	19	2.89E−03	*KANK1*, *RTN4*, *ATP6V1D*, *PTPRK*, *EEF2K*, *ERRFI1*, *CGN*, *TGFBR2*, *ATP6V0A1*, *MVP…*
GO:0043588	Skin development	9	4.97E−03	*PTHLH*, *ALDH3A2*, *ERRFI1*, *YAP1*, *STK4*, *EMP1*, *COL5A2*, *NCOA3*, *DICER1*
GO:0030330	DNA damage response, signal transduction by p53 class mediator	7	1.41E−04	*NDRG1*, *SPRED1*, *PSME3*, *CDKN1A*, *E2F7*, *CASP2*, *HIPK2*
GO:0001890	Placenta development	7	4.74E−04	*TXNRD1*, *ADM*, *CCNF*, *SPP1*, *STK4*, *NDP*, *E2F7*
GO:0031100	Organ regeneration	5	6.05E−05	*ADM*, *TGFBR2*, *CCND1*, *LCP1*, *CDKN1A*
GO:0071456	Cellular response to hypoxia	5	2.26E−03	*HMOX1*, *NPEPPS*, *NDRG1*, *BNIP3*, *HIPK2*
GO:0048002	Antigen processing and presentation of peptide antigen	5	4.35E−02	*CTSD*, *NPEPPS*, *PSME3*, *AP1S1*, *AP1S2*
GO:0055093	Response to hyperoxia	4	2.97E−05	*TXNRD1*, *BNIP3*, *CAV1*, *CDKN1A*
GO:0000188	Inactivation of MAPK activity	4	1.36E−04	*DUSP5*, *SPRED1*, *CAV1*, *DUSP22*
GO:0060443	Mammary gland morphogenesis	4	2.15E−03	*PTHLH*, *TGFBR2*, *CAV1*, *NCOA3*

*GO* Gene Ontology, *ID* identification

(A) The top ten functions enriched for the up-regulated genes. (B) The top ten functions enriched for the down-regulated genes

Among the up-regulated genes, *CCL2* was significantly enriched in the pathway of chemokine signaling pathway (*p* = 1.63E−03). *THBS1* and thrombospondin 2 (*THBS2*) were significantly involved in the pathway of focal adhesion (*p* = 7.54E−03). And the up-regulated genes, such as *THBS1*, *THBS2*, SMAD family member 5 (*SMAD5*) and SMAD family member 6 (*SMAD6*), were significantly enriched in transforming growth factor beta (TGF-beta) signaling pathway (*p* = 4.83E−02) (Table 3A). Meanwhile, the down-regulated transforming growth factor beta receptor II (*TGFBR2*) and *CCND1* were significantly enriched in both the pathways of chronic myeloid leukemia (*p* = 9.85E−03) and pancreatic cancer (*p* = 4.69E−02) (Table 3B).

**Table 3:** The pathways enriched for the differentially expressed genes

KEGG ID	Name	Gene number	*p* value	Gene symbols
(A)				
4062	Chemokine signaling pathway	12	1.63E−03	*PTK2*, *PIK3R2*, *GNG10*, *GNG5*, *RAP1B*, *PIK3R1*, *NRAS*, *IL8*, *GNG12*, *CSK*, *FOXO3*, *CCL2*
4510	Focal adhesion	11	7.54E−03	*PTK2*, *PIK3R2*, *THBS1*, *THBS2*, *RAP1B*, *PPP1CC*, *PIK3R1*, *ITGB1*, *ACTG1*, *FLNB*, *CAV2*
4810	Regulation of actin cytoskeleton	11	1.18E−02	*PTK2*, *PIK3R2*, *CYFIP2*, *PPP1CC*, *PIK3R1*, *NRAS*, *ITGB1*, *GNG12*, *ACTG1*, *CSK*, *ARHGEF6*
4910	Insulin signaling pathway	9	5.22E−03	*PIK3R2*, *PPP1CC*, *PIK3R1*, *NRAS*, *SOCS2*, *PTPN1*, *PYGB*, *CALM2*, *PTPRF*
3013	RNA transport	9	9.27E−03	*PABPC1*, *EIF3A*, *NUP54*, *EIF3G*, *UPF3B*, *NUP155*, *KPNB1*, *NUP37*, *EIF2S3*
4145	Phagosome	8	2.82E−02	*TAP1*, *THBS1*, *THBS2*, *ATP6V1G1*, *ITGB1*, *ACTG1*, *LAMP2*, *DYNC1LI2*
5100	Bacterial invasion of epithelial cells	7	1.24E−03	*PTK2*, *PIK3R2*, *PIK3R1*, *ITGB1*, *RHOG*, *ACTG1*, *CAV2*
5142	Chagas disease (American trypanosomiasis)	7	1.13E−02	*PIK3R2*, *SERPINE1*, *GNA11*, *PIK3R1*, *IL8*, *IFNGR1*, *CCL2*
4722	Neurotrophin signaling pathway	7	3.05E−02	*PIK3R2*, *RAP1B*, *PIK3R1*, *NRAS*, *CALM2*, *CSK*, *FOXO3*
4360	Axon guidance	7	3.28E−02	*PTK2*, *DPYSL2*, *SEMA4F*, *NRAS*, *ITGB1*, *SLIT2*, *EFNA1*
5131	Shigellosis	6	3.01E−03	*ITGB1*, *IL8*, *RHOG*, *ACTG1*, *FBXW11*, *CSK*
5211	Renal cell carcinoma	5	2.45E−02	*PIK3R2*, *RAP1B*, *PIK3R1*, *NRAS*, *EPAS1*
5412	Arrhythmogenic right ventricular cardiomyopathy (ARVC)	5	3.03E−02	*ITGB1*, *DAG1*, *GJA1*, *ACTG1*, *CDH2*
5410	Hypertrophic cardiomyopathy	5	4.62E−02	*TPM3*, *ITGB1*, *DAG1*, *TPM1*, *ACTG1*
4350	TGF-beta signaling pathway	5	4.83E−02	*THBS1*, *THBS2*, *SMAD6*, *ID3*, *SMAD5*
20	Citrate cycle (TCA cycle)	4	5.11E−03	*CS*, *DLD*, *DLAT*, *SDHA*
5144	Malaria	4	3.19E−02	*THBS1*, *THBS2*, *IL8*, *CCL2*
5213	Endometrial cancer	4	3.39E−02	*PIK3R2*, *PIK3R1*, *NRAS*, *FOXO3*
5223	Non-small cell lung cancer	4	3.82E−02	*PIK3R2*, *PIK3R1*, *NRAS*, *FOXO3*
3410	Base excision repair	3	4.23E−02	*POLB*, *PARP1*, *PARP3*
(B)				
4144	Endocytosis	9	4.62E−04	*ASAP2*, *VPS36*, *TGFBR2*, *ASAP1*, *CAV1*, *SH3KBP1*, *EHD1*, *RAB22A*, *DNM3*
4142	Lysosome	7	4.55E−04	*CTSD*, *TPP1*, *ATP6V0A1*, *ABCB9*, *AP1S1*, *AP1S2*, *NEU1*
2010	ABC transporters	4	1.58E−03	*ABCC2*, *ABCC3*, *ABCB9*, *ABCC5*
10	Glycolysis/gluconeogenesis	4	6.56E−03	*ENO2*, *ALDH3A2*, *PGAM1*, *PGK1*
5220	Chronic myeloid leukemia	4	9.85E−03	*TGFBR2*, *CCND1*, *CDKN1A*, *BCL2L1*
561	Glycerolipid metabolism	3	1.98E−02	*ALDH3A2*, *AGPAT9*, *LCLAT1*
5212	Pancreatic cancer	3	4.69E−02	*TGFBR2*, *CCND1*, *BCL2L1*
4966	Collecting duct acid secretion	2	3.85E−02	*ATP6V1D*, *ATP6V0A1*
650	Butanoate metabolism	2	4.67E−02	*AKR1B10*, *HMGCS1*

(A) The pathways enriched for the up-regulated genes. (B) The pathways enriched for the down-regulated genes. Kyoto Encyclopedia of Genes and Genomes, KEGG; identification, ID

### The annotation of DEGs

A total of 54 DEGs were screened as TAGs, including 33 up-regulated and 21 down-regulated genes. Among the 33 up-regulated genes, there were 22 TSGs (such as *THBS1*), 6 oncogenes, and 5 other genes (such as *CCL2*). Meanwhile, there were 13 TSGs, 4 oncogenes (such as *CCND1*), and 4 other genes in the 21 down-regulated genes. Additionally, 9 DEGs were screened as the TFs, including 8 up-regulated and 1 down-regulated genes (Table 4).

**Table 4:** The identified transcription factors (TFs) and tumor associated genes (TAGs) among the differentially expressed genes (DEGs). Tumor suppressed genes, TSGs

DEGs	TF numbers	TFs	TAG numbers	TAGs		
				TSGs	Oncogenes	Others
Up-regulated	1	*KLF12*	33	*BAP1*, *THBS1*, *DKK1*, *PAF1*, *ST13*, *LRP1B*, *PDGFRL*, *ITGB1*, *TPM1*, *GJA1*, *CDH11*, *SLIT2*, *GLIPR1*, *FAT1*, *SOD2*, *FOXO3*, *EFNA1*, *GAS1*, *PTPRF*, *RAD51C*, *CAV2*, *SDHA*	*SET*, *CCNA2*, *AXL*, *NRAS*, *ROS1*, *SCK*	*GTF2F2*, *CTGF*, *FHL2*, *C1QBP*, *CCL2*
Down-regulated	8	*ASCL1*, *ETV4*, *HSF1*, *LMO3*, *PML*, *RUNX3*, *TCF7*, *USF2*	21	*HIPK2*, *YAP1*, *ERRFI1*, *PTPRK*, *KANK1*, *BNIP3L*, *DUSP22*, *SASH1*, *CDKN1A*, *NDRG4*, *ZFHX3*, *NDRG1*, *TGFBR2*,	*BCL2L2*, *NCOA3*, *CCND1*, *CDC25B*	*PTHLH*, *EMP1*, *CAV1*, *GLS*

### PPI network analysis

**Fig. 1:**
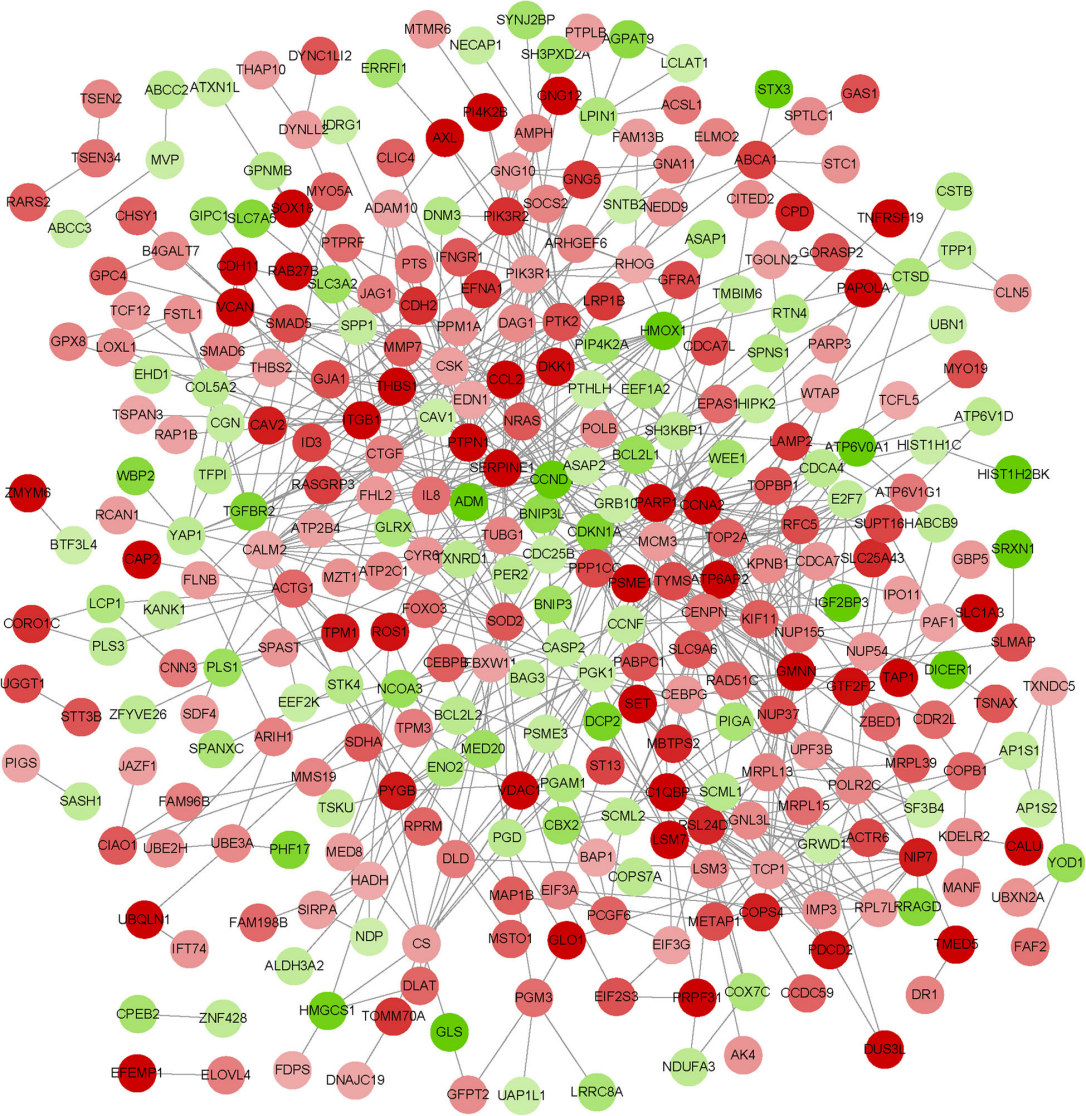
The protein-protein interaction (PPI) network for the differentially expressed genes (DEGs). The red circles represent the up-regulated genes. The green circles indicate the down-regulated genes

The constructed PPI network was consisted of 810 interactions (such as CCND1-THBS1 and THBS1-CCL2) (Fig. 1). Besides, the top 10 % nodes with higher degrees in the PPI network were identified, including CCND1 (degree = 29) and CCL2 (degree = 12) (Table 5).

**Table 5:** The top 10 % DEGs with higher degrees in the protein-protein interaction (PPI) network

Gene	Degree	Gene	Degree	Gene	Degree	Gene	Degree
*CCND1*	29	*SOD2*	19	*CENPN*	16	*KIF11*	15
*PIK3R1*	25	*TYMS*	19	*CAV1*	16	*PTK2*	15
*PGK1*	22	*CDKN1A*	18	*PIK3R2*	16	*EDN1*	14
*NUP37*	22	*PARP1*	18	*CTGF*	15	*CS*	13
*CALM2*	21	*TOP2A*	18	*RFC5*	15	*CCL2*	13
*MCM3*	21	*ITGB1*	18	*NUP155*	15	*RSL24D1*	12
*GMNN*	20	*TCP1*	18	*NRAS*	15	*CDCA7*	12
*CCNA2*	20	*SERPINE1*	17	*NIP7*	15	*BCL2L1*	12

### Transcriptional regulatory network analysis

**Fig. 2:**
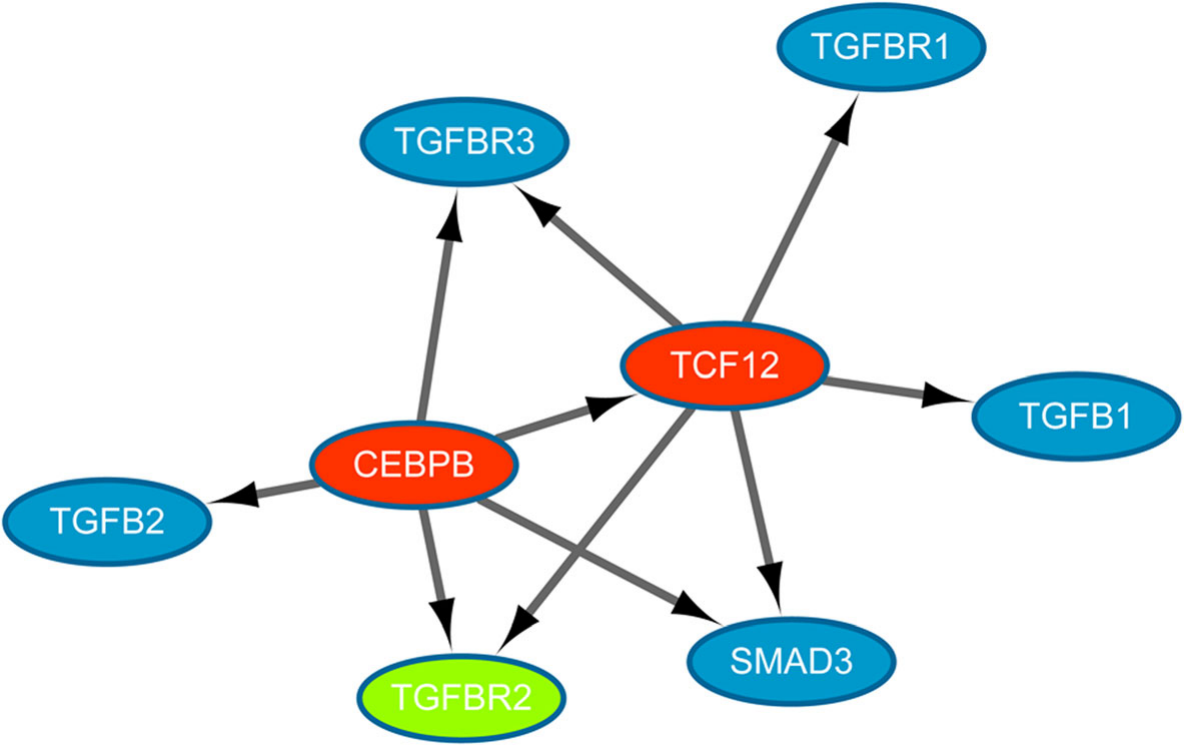
The transcriptional regulatory network involving *CEBPB* and *TGFB1*/*SMAD3*. The red and green nodes represent the up-regulated and down-regulated genes, respectively. The blue nodes stand for non-differentially expressed genes (DEGs). The arrows represent regulatory relationships

For further study, the regulation of *TGFB1*/*SMAD3* by *CEBPB*, the transcriptional regulation interactions related to *TGFB1*/*SMAD3*, and the members of *TGFB* family were screened out from the ENCODE database and the transcriptional regulatory network was visualized by Cytoscape software (Fig. 2). The transcriptional regulation network showed that the *CEBPB* could regulate *SMAD3*, transcription factor 12 (*TCF12*), transforming growth factor beta 2 (*TGFB2*), *TGFBR2*, and *TGFBR3* directly. Additionally, *TCF12* targeted *TGFB1*, *TGFBR1*, *TGFBR2*, *TGFBR3*, and *SMAD3*.

## Discussion

In this study, a total of 529 DEGs were obtained, including 336 up-regulated genes and 193 down-regulated genes. Enrichment analysis indicated that the up-regulated *CCL2* was significantly enriched in the chemokine signaling pathway. Reports have found that chemokine expressed by stromal cells or endogenously produced in glioma cells may play key roles in tumor cell migration, invasion, proliferation, angiogenesis and immune cell infiltration in the tumor mass [26]. The chemokine *CCL2* can promote glioma tumor aggressiveness by promoting attraction of T regulatory cells (which suppress the lymphocyte anti-tumor effector function) and microglial cells (which can reduce the anti-tumor functions and secrete pro-invasive metalloproteinases) [27, 28]. Meanwhile, metalloproteinases can promote the glioma invasion through the detachment of ECM [29]. Besides, results of DEGs annotation showed that *CCL2* was screened out as a TAG. Therefore, we speculated that the increased expression of *CCL2* could promote glioma aggressiveness through the pathway of chemokine signaling.

In addition, some up-regulated genes (such as *THBS1*, *THBS2*, *SMAD5*, and *SMAD6*) were significantly enriched in the TGF-beta signaling pathway in our study. Recently, it has been reported that the *TGFB* is a key factor in controlling migration, invasion and angiogenesis in glioblastoma and induces profound immunosuppression [30]. Besides, the *THBS1* (belonging to thrombospondin family), which is referred as a *TGFB* activating protein, induces the glioma invasion [31]. THBS1 is a powerful anti-angiogenesis protein in glioblastoma [32]. These suggested that *THBS1* might play a key role in regulating the angiogenesis in glioma. As another member of thrombospondin family, *THBS2* may be a potential inhibitor of tumor growth and angiogenesis [33]. Moreover, it has been shown that *THBS2* can function as an endogenous inhibitor of angiogenesis through directly affecting endothelial cell migration, proliferation, survival, and apoptosis [34]. In our study, we also found that *THBS1* and *THBS2* were significantly involved in the pathway of focal adhesion. Previous study reported that focal adhesion can suppress the migration and metastasis of tumor cells [35]. Therefore, we speculated that *THBS1* and *THBS2* could regulate angiogenesis and invasion in glioma via TGF-beta signaling pathway and focal adhesion pathway. Former researches have shown that *SMAD6* is an inhibitor of *TGFB* signaling and blocked the phosphorylation of receptor-regulated *SMADs* (such as *SMAD5*) in the cytoplasm [36]. As a result, we assumed that *SMAD5* and *SMAD6* might affect glioma by regulating the *TGFB* signaling. In the PPI network, *THBS1* could interact with CCL2, to some extent, indicating that *THBS1* might play key roles in glioma through regulating *CCL2*. Consequently, *THBS1*, *THBS2*, *SAMD5* and *SMAD6* could be key factors involved in the *CEBPB*-silenced glioma.

Moreover, CCND1, as a member of the cyclin family, possessed the highest degree in the PPI network. Cyclins can modulate tumor cell cycle through alterations in cyclin-dependent kinase activity [37]. What’s more, researchers have discovered that overexpression of *CCND1* can elevate the proliferation and invasion potential of human glioblastoma cells [38]. In the PPI network, we also found that CCND1 had interaction with THBS1, suggesting that *CCND1* could be involved in regulating proliferation and invasion of glioma via interacting with *THBS1*.

*TGFBR2* plays a key role in *TGFB* signal propagation via activating *TGFBR1* and the phosphorylation of SMAD proteins [39]. Moreover, silencing of *TGFBR2* can abolish *TGFB*-induced invasion and migratory responses of glioblastoma in vitro [40]. In our study, we also discovered that the up-regulated *TCF12* could regulate *TGFB1* and *SMAD3*, indicating that *CEBPB* might regulate *TGFB1* and *SMAD3* through *TCF12*. Previous studies have shown that *TGFB1*/*SMAD3* can promote tumor cell migration, invasion and metastasis through inducing epithelial-mesenchymal transition [41, 42]. What is more, *TCF12* has been found to suppress the expression of E-cadherin, which can lead to the metastasis of tumor cells [43]. Therefore, we assumed that *CEBPB* might regulate *TGFBR2* and *SMAD3* through *TGF-β1*/*SMAD3* signaling pathway in glioma, and *CEBPB* could also affect metastasis of glioma by regulating *TCF12*. However, in our study, *TGFB1* and *SMAD3* were not significantly expressed, which might due to the relatively short time for *CEBPB* silencing. In our further research, the regulation of *CEBPB* on *TGFB1*/*SMAD3* will be studied with *CEBPB-*silenced for a relatively long time.

## Conclusions

We conducted a comprehensive bioinformatics analysis to identify genes which may be correlated with *CEBPB*-silenced glioma. A total of 529 DEGs were identified in the normal glioma cells compared with the *CEBPB*-silenced glioma cells. Besides, The identified DEGs, such as *TCF12*, *TGFBR2*, *CCL2*, *THBS1*, *THBS2*, *SMAD5*, *SMAD6*, and *CCND1*, might play important roles in the progression of glioma via the regulation of *CEBPB*. However, further researches are still needed to unravel their action mechanisms in glioma.

## Abbreviations

BH: Benjamini-Hochberg
DEGs: Differentially expressed genes
ECM: Extracellular matrix
ENCODE: Encyclopedia of DNA Elements
FDR: False discovery rate
GEO: Gene Expression Omnibus
KEGG: The Kyoto Encyclopedia of Genes and Genomes
MSC: Mesenchymal stem cells
PPI: Protein-protein interaction
TAG: Tumor-associated gene
TF: Transcription factor
TSG: Tumor suppressor gene
